# Demographic, clinical, laboratory and treatment characteristics of spondyloarthritis patients with and without acute anterior uveitis

**DOI:** 10.1590/S1516-31802012000300002

**Published:** 2012-07-12

**Authors:** Marcelo Gehlen, Kelly Cristina Regis, Thelma Larocca Skare

**Affiliations:** I MD, PhD. Mentor of the Rheumato-ophthalmology Service, Hospital Universitário Evangélico de Curitiba, Curitiba, Paraná, Brazil.; II MD. Resident in the Rheumatology Service, Hospital Universitário Evangélico de Curitiba, Curitiba, Paraná, Brazil.; III MD, PhD. Head of the Rheumatology Service, Hospital Universitário Evangélico de Curitiba, Curitiba, Paraná, Brazil.

**Keywords:** Spondylarthropathies, Spondylitis, ankylosing, Uveitis, Prognosis, Arthritis, psoriatic, Espondiloartropatias, Espondilite anquilosante, Uveíte, Prognóstico, Artrite psoriásica

## Abstract

**CONTEXT AND OBJECTIVE:**

Acute anterior uveitis is a common extra-articular manifestation in spondyloarthritis patients. The aim of this study was to compare demographic, clinical, laboratory and treatment data among spondyloarthritis patients with and without acute anterior uveitis.

**DESIGN AND SETTING:**

This was a cross-sectional analytical study at the Rheumatology Outpatient Clinic of the Evangelical University Hospital, Curitiba, Brazil.

**METHODS:**

Spondyloarthritis patients with without acute anterior uveitis were compared regarding demographic data, spondyloarthritis subtype, peripheral arthritis, enthesitis, disease activity, functional index, physical examination, radiological involvement, HLA-B27 and treatment.

**RESULTS:**

Presence of acute anterior uveitis was not found to have any relationship with functional index, degree of radiological involvement, peripheral arthritis or enthesitis. Acute anterior uveitis showed a negative association with skin manifestations (P = 0.04) and a trend towards higher disease activity (P = 0.06).

**CONCLUSION:**

In the study sample, it could not be shown that AAU had any association with the functional and radiological prognoses. The patients with spondyloarthritis with and without acute anterior uveitis did not differ clinically except for a higher proportion of ankylosing spondylitis and smaller presence of skin involvement in those with uveitis.

## RESUMO

**CONTEXTO E OBJETIVO:**

Uveíte anterior aguda é uma manifestação extra-articular comum em pacientes com espondiloartrite. O objetivo deste estudo foi comparar achados demográficos, clínicos, laboratoriais e de tratamento em pacientes com espondiloartrite com e sem uveíte anterior aguda.

**TIPO DE ESTUDO E LOCAL:**

Este é um estudo transversal analítico realizado no Ambulatório de Reumatologia do Hospital Universitário Evangélico de Curitiba, Brasil.

**MÉTODOS:**

Pacientes com espondiloartrite com e sem uveíte anterior aguda foram comparados quanto a dados demográficos, subtipo de espondiloartrite, artrite periférica, entesite, atividade da doença, índice funcional, exame físico, envolvimento radiológico, HLA-B27 e tratamento.

**RESULTADOS:**

Não se encontrou associação entre presença de uveíte anterior aguda e índice funcional, grau de envolvimento radiológico, artrite periférica e entesite. Encontrou-se uma associação negativa de uveíte anterior aguda com manifestações de pele (P = 0,04) e uma tendência para maior atividade de doença (P = 0,06).

**CONCLUSÃO:**

Na amostra estudada, não foi possível demonstrar associação entre uveíte anterior aguda no prognóstico funcional e radiológico. Pacientes com espondiloartrite com e sem uveíte anterior aguda não diferem clinicamente exceto por uma maior proporção de espondilite anquilosante e uma presença menor de envolvimento cutâneo naqueles com uveíte.

## INTRODUCTION

Spondyloarthritis (SpA) comprises at least five diseases: ankylosing spondylitis (AS), psoriatic arthritis (PsA), reactive arthritis (ReA), arthritis of inflammatory bowel disease (AIBD), juvenile spondylitis (jSpA) and undifferentiated spondyloarthritis (uSpA). These diseases all share a common clinical pattern and pathophysiological mechanisms.^1^ They may present not only spinal and peripheral joint involvement but also a series of extra-articular manifestations such as uveitis, enthesitis, skin lesions, apical lung fibrosis, valvular aortic incompetence and cardiac blocks.^2^ They are also related to the presence of the major histocompatibility complex (MHC) class 1, in the HLA-B27 form.^2^

Uveitis is considered to be one of the most common extra-articular manifestations of SpA, second only to skin lesions, and its prevalence may vary according to the genetic background of the study population.^3^ It is usually acute, affects the anterior uveal tract and is unilateral and self-limited, but it may recur and complicate with glaucoma or cystoid macular edema, thereby resulting in a sight-threatening complication that may diminish the patient’s quality of life.^4^

The prevalence of acute anterior uveitis (AAU) also varies according to the subtype of SpA studied. A study done on a Brazilian population has shown prevalences of 14.5% for AS and 8.8% for uSpA.^5^ AS is considered to be the disease with the highest prevalence of AAU, and such manifestations are less common in ReA and PsA.^6^

## OBJECTIVE

To compare demographic, clinical, laboratory and treatment data among SpA patients with and without AAU.

## METHODS

This was an analytical cross-sectional study that was approved by the local Research Ethics Committee. All the participants gave their signed consent. One hundred and two consecutive patients from our Rheumatology Unit were included, and all of them fulfilled the European Spondylarthropathy Study Group (ESSG) criteria for SpA.^7^ This number of patients represented the total number of patients with SpA over a one-year period who agreed to participate in the study.

The patients answered the Bath Ankylosing Spondylitis Disease Activity Index (BASDAI) and Bath Ankylosing Spondylitis Functional Index (BASFI) questionnaires and underwent a physical examination for Bath Ankylosing Spondylitis Metric Index (BASMI) evaluation. BASDAI is an instrument that measures a disease activity index by means of visual analogue scales representing five variables: spinal pain, peripheral joint pain, pain at enthesopathy sites, morning stiffness and fatigue. BASDAI values range from zero to ten and if over four, this is considered suggestive of high disease activity.^7^ BASFI is a functional index, evaluated through ten questions about daily activities. Its scores also range from zero to ten (where zero points is the best possible performance and ten is the worst).^8^ Both BASDAI and BASFI are questionnaires that have been validated for the Portuguese language.^9^ BASMI is a measurement of physical findings from physical examination of SpA patients, in which the Schober test, degree of cervical rotation, lateral spinal flexion and intermalleolar and tragus-wall distance are taken into account. BASMI scores range from zero (no alteration at physical examination) to ten (severe physical impairment).^10^

The patients’ medical files were reviewed to obtain data on demographic characteristics, diagnoses of disease subtypes, disease duration, uveitis occurrence, enthesopathy, peripheral arthritis, electrocardiograms and HLA-B27. Histories of uveitis were taken into consideration when diagnosed by an ophthalmologist. Radiographic films on the sacroiliac joints, lumbar and cervical spine and hips were available for 72 patients, and the Bath Ankylosing Spondylitis Radiological Activity Index (BASRI) score was measured in relation to these patients. BASRI measures the degree of cervical and lumbar spine, sacroiliac joint and hip involvement seen on X-ray images in SpA patients. It can range from zero (no abnormality) to 16 (maximum abnormality).^11^ All the X-ray readings were done by the same rheumatologist.

The data obtained were studied through contingency and frequency tables. To evaluate associations relating to nominal data, the chi-square and Fisher tests were used. To evaluate associations relating to numerical data, the Mann-Whitney and unpaired t tests were used. Calculations were done with the aid of the GraphPad Prism software, version 4.0. The significance level adopted was 5% and the power of the study sample was 75%.

## RESULTS

Among the 102 patients (42 females and 60 males), the mean age at diagnosis was 43.7 ± 12.2 years; the mean age at first musculoskeletal symptoms was 31.6 ± 11.3 years; and the median disease duration was four years. In this sample, 28.2% were Afro-descendants (blacks and mulattos) and 71.7% were Caucasians. The most common disease subtype was AS, which was present in 59/102 patients (57.8%). Other subtypes found were: PsA in 16/102 (15.6%), uSpA in 13/102 (12.7%); ReA in 6/102 (5.8%); JSpA in 5/102 (4.9%) and AIBD in 3/102 (2.9%). Non-steroidal anti-inflammatory drugs were used in 86.3%; glucocorticoids in 42.15%; sulphasalazine in 43.1%, methotrexate in 44.1% and anti-tumor necrosis factor alpha drugs in 21.5%.

HLA-B27 was positive in 39/57 patients (68.4%). BASDAI ranged from 0 to 9.15 (mean: 4.16 ± 2.45), BASFI from 0 to 10 (mean: 4.40 ± 3.04), BASMI from 0 to 9 (median: 2) and BASRI from 0 to 16 (mean: 6.57 ± 4.62). In 71/102 (69.6%), there was peripheral joint involvement; in 55/102 (53.9%), there was enthesopathy; 16 (15.65) had skin involvement; one (0.9%) had aortic valvular lesion; one (0.9%) had apical lung fibrosis. An electrocardiogram was produced for 59 patients, and nine (15.2%) of them presented cardiac block: one patient had second-degree A-V block and all the others had right bundle branch block.

Uveitis was found in 22/102 (21.5%). In 45.4% (10/22), the patients had had just one episode; in 27.2% (6/22), from two to five episodes; and in 27.2% (6/22) more than five episodes.

Comparing SpA subtypes in patients with and without uveitis, we found that AS patients had had more episodes of uveitis than those with other forms of SpA (P = 0.002). Table 1 shows the evaluation of clinical, functional and demographic variables in relation to the presence of uveitis and Table 2, the treatment.

**Table 1.** Comparison of demographic, clinical and radiological data in spondyloarthritis patients with and without uveitis (n = 102)

	With anterior acute uveitis n = 22	Without anterior acute uveitis n = 80	P
Gender (female/male)	7/15	35/45	0.83
Caucasian ethnic background	71.4%	71.7%	0.97
Mean age (years)	47.50 ± 11.20	42.73 ± 12.36	0.10
Mean age at first symptom (years)	29.65 ± 11.48	29.67 ± 11.30	0.36
Median disease duration (years)	5	4	0.35
Tobacco exposure (present or past)	38.4%	31.2%	0.64
Cardiac blocks (shown on electrocardiogram)	26.6%	22.7%	0.21
Peripheral arthritis	54.5%	73.7%	0.33
Enthesitis	45.5%	56.2%	0.36
Skin lesions	0	21.2%	0.04
BASDAI (mean)	4.39 ± 2.39	3.25 ± 2.51	0.063
BASFI (mean)	3.37 ± 3.32	4.67 ± 2.92	0.08
BASMI (median)	2	2	0.59
BASRI (mean)^*^	6.35 ± 4.50	7.26 ± 5.02	0.46
HLA-B27^†^	66.6%	69.0%	0.86

BASDAI = Bath Ankylosing Spondylitis Disease Activity Index; BASFI = Bath Ankylosing Spondylitis Functional Index; BASMI = Bath Ankylosing Spondylitis Metric Index; BASRI = Bath Ankylosing Spondylitis Radiological Activity Index. ^*^Data available on 72 patients; ^†^Data available on 57 patients.

**Table 2.** Treatment for spondyloarthritis patients with and without uveitis (n = 102)

	With anterior acute uveitis n = 22	Without anterior acute uveitis n = 80	P
Non-hormonal anti-inflammatory drugs	77.27%	88.75%	0.6
Glucocorticoids	40.9%	42.5%	0.85
Sulphasalazine	54.5%	40%	0.22
Methotrexate	18.1%	51.2%	0.007
Anti-tumor necrosis factor alpha	22.7%	21.2%	1.00

## DISCUSSION

The relationship between AAU and SpA is well established and this may be the first clinical sign of the disease. However, the pathogenetic link between ocular and skeletal disease remains undetermined.^3^

In the present series of 102 SpA patients, we documented a prevalence of AAU of 21.5% and that this complication was most common in AS patients, but no relationship between history of AAU and the functional impairment (BASFI), radiological progression (BASRI), joint mobility (BASMI) or extra-articular disease could be found. The rate of uveitis occurrence found in our sample was consistent with the range cited in the literature: between 18 and 25%.^3^

Gran et al.^2^ studied disease outcome in 100 patients and found that the subgroup of patients with AAU had a greater functional reduction in daily activities, but their sample was made up of ankylosing spondylitis patients only. Robertson et al.^12^ also studied AS patients and detected a more rapid deterioration of function, as measured by BASFI, in those with AAU. A Chinese study^13^ on 140 AS patients found that the subgroup of patients with AAU had greater disease activity and poorer functional ability. Similarly to what we found in the present study, Ward et al.^14^ could not detect differences in functional status between AS patients with and those without AAU, in a study on 326 patients.

Chen et al.^13^ found an association between AAU and BASDAI. In the present study, no significant relationship between higher disease activity in AAU and SpA could be demonstrated statistically although a trend was found (P = 0.06). It is possible that the study sample did not have enough statistical power to demonstrate this finding. The sample of 22 patients with AAU gave a power of around 75% to detect a difference of 2.0 (taking into consideration the minimum significant difference) in BASDAI. In addition, the group with AAU was receiving less methotrexate because most of them were AS patients, and this medication has no proven effect in patients with predominance of axial involvement. The value of methotrexate in cases of AAU and SpA is controversial and based on small studies,^15^ since most studies on uveitis have not classified the uveitis treatment according to etiology.^16^^,^^17^

A negative relationship was found between skin disease (psoriasis) and the presence of AAU. Uveitis in psoriatic arthritis cases is considered to be less common than in other forms of SpA. A systematic analysis on the literature found in the Medline database^6^ from 2004 to 2006 covering 29,877 patients showed that PsA had the smallest prevalence of uveitis, losing only to uSpA.

In the present study, no association could be found between occurrences of uveitis, peripheral arthritis and enthesitis, unlike in some other reports.^5^ This variation can be explained by the mixed nature of the study populations, which have encompassed all subtypes of SpA. PsA has some clinical peculiarities, with a higher proportion of peripheral involvement than seen in AS cases. Since most PsA cases do not have uveitis,^6^ the lack of association between ocular findings and peripheral musculoskeletal symptoms can be explained by participation of this subtype.

## CONCLUSION

Concluding, AAU is more common in AS patients and less common in those with skin disease. The overall prevalence of AAU in the group of SpA patients was 21.5%. A negative association was found with skin lesions, but it could not be shown that AAU had any influence on the functional and radiological outcomes among this sample of SpA patients.
